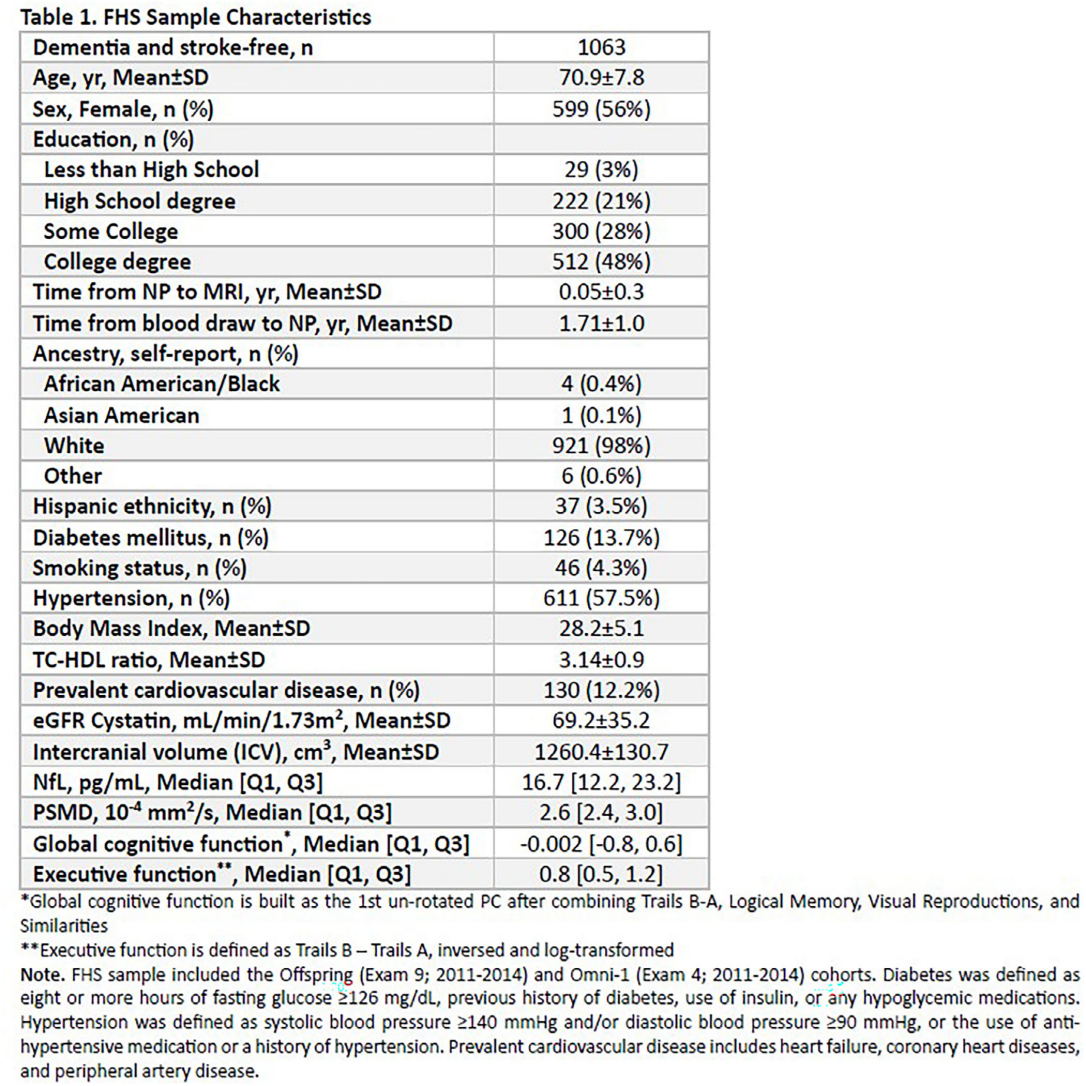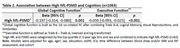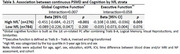# Improving VCID Risk Stratification: A NfL and PSMD Multi‐Biomarker Approach

**DOI:** 10.1002/alz70856_106005

**Published:** 2026-01-08

**Authors:** Alison M. Luckey, Rebecca Bernal, Alexa S Beiser, Jayandra Jung Himali, Hugo J. Aparicio, Pauline Maillard, Sudha Seshadri, Claudia L Satizabal

**Affiliations:** ^1^ Glenn Biggs Institute for Alzheimer's & Neurodegenerative Diseases, University of Texas Health San Antonio, San Antonio, TX, USA; ^2^ The Framingham Heart Study, Framingham, MA, USA; ^3^ Department of Biostatistics, Boston University School of Public Health, Boston, MA, USA; ^4^ Department of Neurology, Boston University Chobanian & Avedisian School of Medicine, Boston, MA, USA; ^5^ Department of Neurology and Center for Neuroscience, University of California, Davis, Davis, CA, USA; ^6^ Glenn Biggs Institute for Alzheimer's & Neurodegenerative Diseases, University of Texas Health Science San Antonio, San Antonio, TX, USA

## Abstract

**Background:**

Neurofilament Light (NfL) is a broad biomarker of neuroaxonal injury elevated in neurological diseases, including cerebral small vessel disease (cSVD). However, NfL's non‐specificity limits its effectiveness in assessing susceptibility/risk for Vascular Contributions to Cognitive Impairment and Dementia (VCID). To improve VCID risk stratification, we propose combining NfL with Peak‐Width of Skeletonized Mean Diffusivity (PSMD), a *specific* neuroimaging biomarker of white matter microstructural damage. Prior research shows NfL and PSMD, individually, are strongly associated with worse cognition. This study aims to (1) provide proof‐of‐concept validation for a NfL‐PSMD multi‐biomarker approach, and (2) evaluate a two‐step strategy using NfL as an initial screening tool, followed by PSMD measurement to further delineate the presence of cSVD‐VCID features.

**Method:**

Participants (*N* = 1063) from the Framingham Heart Study Offspring and Omni‐1 cohorts, with NfL, neuroimaging, and cognitive data were included (Table 1). NfL was measured in plasma, and PSMD was derived from magnetic resonance diffusion‐weighted imaging. Executive and global cognitive function were assessed using a neuropsychological battery. NfL and PSMD were categorized by the top quartile and combined to indicate heightened VCID risk. Linear regression models examined the association between high NfL‐PSMD and cognition, adjusting for age, age^2^, sex, education, renal function (eGFR), total intracranial volume, time difference between blood draw/MRI and cognitive assessment, and cohort. We then stratified by high and low NfL to assess whether PSMD was differentially associated with cognition.

**Result:**

High NfL‐PSMD was significantly associated with worse executive (Beta[95% confidence interval], ‐0.059[‐0.093, ‐0.025], *p* = 0.001) and global cognitive function (‐0.197[‐0.314, ‐0.080], *p* <0.001) (Table 2). After stratification by NfL, higher PSMD was significantly associated with poorer executive (‐0.108 [‐0.180, ‐0.036], *p* = 0.003) and global cognitive function (‐0.451[‐0.684, ‐0.217], *p* <0.001) among those within the high NfL strata (Table 3).

**Conclusion:**

The NfL‐PSMD multi‐biomarker approach identifies persons with heightened VCID risk and worse cognition. Further, NfL modifies the association between PSMD and cognition only among those with high NfL. This proposed two‐step approach may offer a cost‐effective tool for selecting participants for prevention trials of VCID. Ongoing analyses are exploring the added specificity of the NfL‐PSMD multi‐biomarker, with additional validation studies in larger, diverse samples underway.